# *Chlorella vulgaris* Extract-Decorated Gold Nanoparticle Hybridized Antimicrobial Hydrogel as a Potential Dressing

**DOI:** 10.3390/gels9010011

**Published:** 2022-12-25

**Authors:** Ruiying He, Dong Zhou, Lan Xiao, Yulin Li

**Affiliations:** 1 College of Chemistry and Chemical Engineering, Hubei University, Wuhan 430062, China; 2 The Key Laboratory for Ultrafine Materials of Ministry of Education, State Key Laboratory of Bioreactor Engineering, Engineering Research Center for Biomedical Materials of Ministry of Education, School of Materials Science and Engineering, East China University of Science and Technology, Shanghai 200237, China; 3 School of Chemistry and Chemical Engineering, Nanchang University, Nanchang 330031, China; 4 School of Mechanical, Medical and Process Engineering, Center of Biomedical Technology, Queensland University of Technology, Brisbane, QLD 4059, Australia; 5 Australia-China Centre for Tissue Engineering and Regenerative Medicine, Queensland University of Technology, Brisbane, QLD 4059, Australia

**Keywords:** chlorella extracts, hydrogel, gold nanoparticles, antibacterial

## Abstract

This study reports a novel design of a moisturizing and antimicrobial hydrogel with injectable properties, using a green solvent (glycerol) as a cross-linking agent and gold nanoparticle reduced by Chlorella extract as an antimicrobial approach. We have synthesized gold nanoparticles (AuNPs) with environmentally friendly and bio-safe properties using Chlorella aqueous extracts (AuNPs@Chlorella). Characterization of the nanoparticles by ultraviolet-visible spectroscopy (UV-Vis), Fourier transform infrared spectroscopy (FTIR), Raman spectrum, and transmission electron microscope (TEM) confirmed that spherical AuNPs with the particle size of 10–20 nm were successfully synthesized. An analysis of the enhancement of the stability of gelatin hydrogels by the addition of glycerol and AuNPs was performed by rheometry. In addition, we also used *Staphylococcus aureus* (*S. aureus*) and *Escherichia coli* (*E. coli*) to confirm the good antibacterial activity. Therefore, the as-prepared gelatin–glycerol hydrogels containing AuNPs@Chlorella are most likely promising alternatives for wound healing dressings.

## 1. Introduction

The skin is humans’ first defense against UV radiation, microorganisms, pollutants, and chemicals [1]. Damage to skin integrity caused by disease or external factors can cause varying degrees of impact, such as wound swelling, inflammation, ulceration, and in severe cases, amputation or even death [2]. Wounds are divided into acute wounds and chronic wounds. The healing period for acute wounds is 8 to 12 weeks, and chronic wounds are usually more than 12 weeks. Wound healing is a dynamic and highly complex process that typically consists of four phases: coagulation and hemostasis, inflammation, proliferation, and wound remodeling [3]. According to reports, about 6% of the world’s population suffers from chronic wounds, costing almost USD 20 billion a year in medical care [4]; in the United States, 3% of people over the age of 65 have wounds at any one time, making wound care a burden in national health care systems [5].

Hydrogel, as a material with a three-dimensional mesh structure formed by cross-linking natural or synthetic polymers, has attracted the attention of many scientists for its unique properties of holding large amounts of water to provide a moist environment to the wound site and absorbing the wound exudate without dissolving itself [6]. In recent years, scientists have been working on multi-functional research around hydrogels, aiming to make hydrogels an ideal wound dressing. Gelatin, a peptide polymer extracted from collagen, has a wide range of applications in the food, cosmetic and pharmaceutical industries [7]. Meanwhile, gelatin is widely used in developing wound dressings due to its good biocompatibility, degradability, low antigenicity, controlled biodegradability, and capability to stimulate cell adhesion and growth [8]. When the temperature is below 35 °C, gelatin sols turn into gels, which means that gelatin-based hydrogels have deficiencies when applied to the skin (37 °C). To solve this problem, chemical cross-linking agents (e.g., glutaraldehyde, carbodiimide, and genipin) are generally used to elevate gelatin hydrogels’ phase transition temperature, which causes potential toxicity [9].

Glycerol is a green solvent with a long history of clinical application [10]. Glycerol molecules are widely used as moisturizers and anti-freeze agents to improve the environmental tolerance of hydrogels [11] by forming a large number of hydrogen bonds between glycerol molecules and water molecules, which hinder the evaporation of free water and the formation of ice crystals in hydrogels. At the same time, glycerol is also a plasticizer [12]. When added to hydrogels, it can compensate for the lack of mechanical properties of natural polymer hydrogels to better match the expectations of ideal dressings.

Bacterial presence has always been a problem in wound management [13]. Previously, many researchers achieved the antibacterial effect by loading antibiotics, metal ions, or antimicrobial peptides, which are associated with drawbacks: Antibiotics are prone to resistance, metal ions are potentially toxic and lost too quickly, and antimicrobial peptides are expensive and complicated to prepare [14], which are not the best choice. In recent years, there has been growing interest in gold nanoparticles (AuNPs) synthesized using natural medicinal plants as an antibacterial approach [15]. It is more promising for biomedical applications due to the biosafety and low cost of the AuNPs synthesized by using such a method green, compared to traditional reducing agents (sodium citrate, sodium borohydride) [16]; meanwhile, the plant extracts (polyphenolic and aliphatic components) have antibacterial [17], antioxidant, and anti-inflammatory effects, expanding the scope of AuNPs applications [18].

In this paper, we designed a novel hydrogel dressing (CGGel hydrogel) using green solvent (glycerol) as a cross-linking agent and nanoparticles synthesized from *Chlorella vulgaris* extract as an antimicrobial agent to expand the biomedical application of gelatin-based hydrogels. CGGel hydrogels were shown to have water retention, injectability, good antimicrobial properties, stability, and phase transition temperature by physical cross-linking (massive hydrogen bonding). Meanwhile, to our knowledge, yet to be investigated is the potential application of AuNPs synthesized with Chlorella extracts loaded onto gelatin–glycerol thermosensitive hydrogels as skin dressings.

## 2. Results and Discussion

### 2.1. Synthesis and Characterization of AuNPs

#### 2.1.1. UV-Vis Spectroscopy Characterization of AuNPs@Chlorella

**Figure 1:**
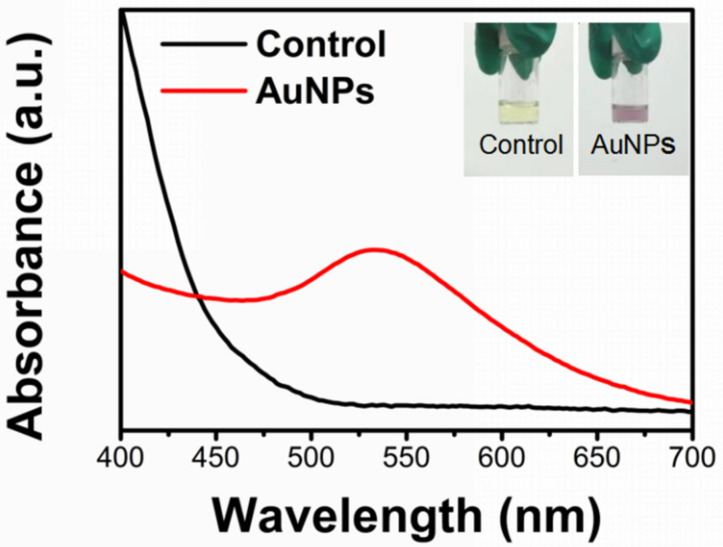
General morphology and UV-vis results of AuNPs synthesized with Chlorella.

Based on the previous work [19], AuNPs@Chlorella were synthesized using Chlorella aqueous extract as a bio-reductive stabilizer. The main components of Chlorella extract were amino acids, proteins, polysaccharides, vitamins, etc. We observed a reduction of Au^+3^ to its metallic state, Au^0^, via the reaction solution from yellow to violet, demonstrating the formation of AuNPs (as shown in Figure 1). AuNPs have specific surface plasmon resonance (SPR) in the visible range. Further verification was performed using UV-Vis spectroscopy (Figure 1), which shows the highest peak at 534 nm^−1^, consistent with the characteristics of AuNPs.

#### 2.1.2. Characterization of the Qualitative Structure of AuNPs@Chlorella

**Figure 2:**
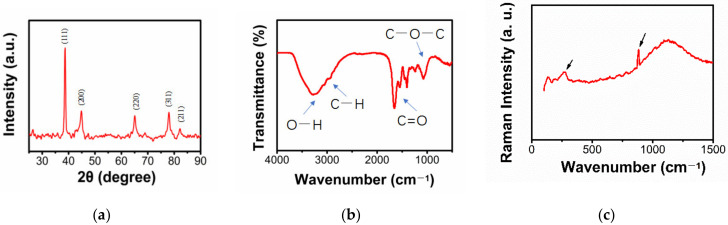
(**a**) XRD pattern of obtained AuNPs@Chlorella. (**b**) FTIR results of AuNPs@Chlorella synthesized. (**c**) Raman spectra of the biostabilized colloidal AuNPs@Chlorella.

The XRD was carried out for further demonstration. As shown in Figure 2a, the results obtained correspond to reflections at 38.201°, 44.401°, 64.601°, 77.59°, and 81.756°2θ for all samples in the (111), (200), (220), (311), and (211) planes, respectively, demonstrating the formation of crystalline GNPs.

It was reported that the formation of metal nanoparticles has capping peptides with amide-I and amide-ii bonds (bimolecular crystallization, 1635 cm^−1^) [20], so we performed an FTIR study that demonstrated the presence of the 1635 cm^−1^ main peak. Meanwhile, we also identified the more characteristic peaks shown in Figure 2b: a broad and large peak at 3300 cm^−1^ is typical of the -OH stretching bands, a strong absorbing -C=O peak at the range of 1700 -1600 cm^−1^, and the appearance of small peaks in the range of 2955–2855 cm^−1^, which are -CH_3_ and methylene -CH_2_ groups. It is considered that the Chlorella extract components (pigments, polysaccharides, and moieties of peptides and proteins) involved in the Au^+3^ reduction of GNPs are present on the surface of GNPs through electrostatic interaction.

Raman spectroscopy was used to observe the vibrations of the material. As shown in Figure 2c, the synthesized AuNPs should be observed to have typical absorption bands in the range of 300–900 cm^−1^ [21]. The vibrational peaks exhibited near 326 cm^−1^ and 950 cm^−1^ are attributed to Au-O vibrations and O-O vibrations, demonstrating the synthesis of stable colloids.

#### 2.1.3. Characterization of the Morphology and Size of AuNPs@Chlorella

**Figure 3:**
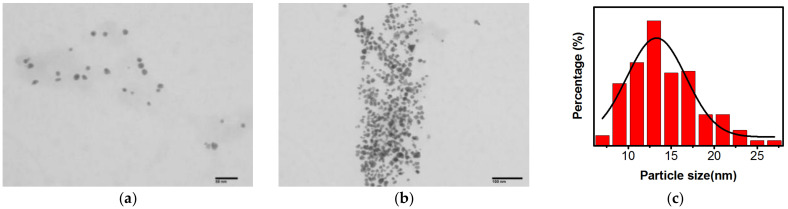
TEM images of AuNPs@Chlorella with different magnifications (**a**) (Scale bar: 50 nm) and (**b**) (Scale bar: 100 nm). (**c**) The particle size distribution of AuNPs@Chlorella.

The shape and size of the nanoparticles are highly crucial for specific applications. Hence, transmission electron microscopy (TEM) was used to further confirm the size and morphological characteristics of AuNPs@Chlorella. As shown in Figure 3a, the nanoparticles show uniform size (diameter around 12 nm) and spherical shape. To further confirm the AuNP size, ImageJ was used to analyze an extensive range of AuNPs (Figure 3b,c). Further analysis of the images showed that the particle size was distributed in 8–20 nm (Figure 3b,c). 

### 2.2. Preparation and Characterization of Hydrogels

#### 2.2.1. Gel Time of CGGel Gels

**Figure 4:**
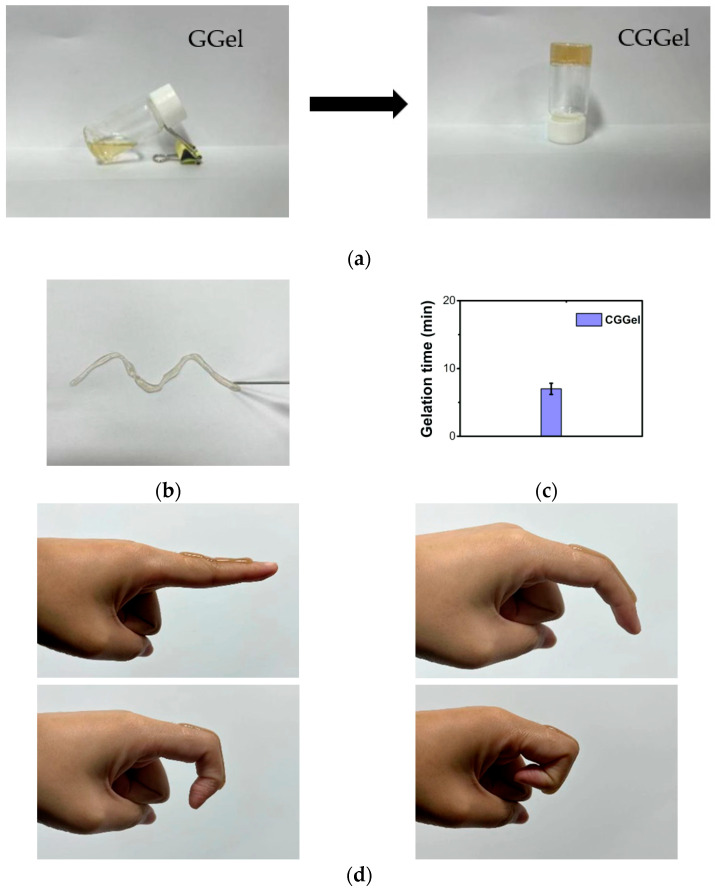
(**a**) Optical images of the gelation progress of the CGGel hydrogel. (**b**) The photograph showed the injectability of the CGGel hydrogel. (**c**) Gelation time of the CGGel hydrogel. (**d**) The CGGel hydrogel adhered to the skin of the author.

Gelatin, a natural protein with amino acid sequences such as Arg-Gly-Asp (RGD), is widely used in commercial medical dressings. The triple helix structure and reactive groups of gelatin offer more possibilities for its application. It is well known that gelatin gelation temperature is below 37 °C, a disadvantage of gelatin-based hydrogels [22]. When glycerol and AuNPs@Chlorella were added to a vial containing gelatin solution (GGel) and stirred well to form CGGel hydrogel (AuNPs@Chlorella–Glycerol–Gelatin hydrogel), the conversion of the sol to a gel was observed after a period of time (Figure 4a). Subsequently, the CGGel hydrogel did not flow when the vial was inverted. We consider that it is the cross-linking by hydrogen bonding of the reactive groups (amino, carboxyl, hydroxyl) in gelatin with glycerol (hydroxyl) and AuNPs@Chlorella (amino, carbonyl, carboxyl, hydroxyl) in large amounts that raises the gelation transition temperature.

In addition, gelatin is a temperature-sensitive material with the potential for injectable properties [23]. Therefore, by placing CGGel in a syringe, the gel became soluble within 3 min at 50 °C. Subsequently, we injected it in an open environment (Figure 4b). Surprisingly, the solution solidified within seconds and stuck firmly to the paper, which is very promising for medical applications. Furthermore, we determined the gelation time (Figure 4c), which is crucial for in situ forming of hydrogels: CGGel hydrogels showed a gelation time of around 7 min, while Gel and GGel hydrogels could not gel at 37 °C.

#### 2.2.2. Adhesivity of CGGel Gels

Adhesion properties are also significant for medical applications [24]. Therefore, to test its adhesion properties, CGGel hydrogel was injected through a syringe onto the authors’ skin (finger joints). It was found that the sol rapidly formed a gel and adhered tightly to the skin, following which it remained stable and unbroken when we cyclically bent the finger at 45°, 90°, and 135° (Figure 4d). The reason for that is the formation of strong and reversible hydrogen bonds between the reactive groups in the gelatin chain and the groups on the skin.

#### 2.2.3. Stability of CGGel Gels

**Figure 5:**
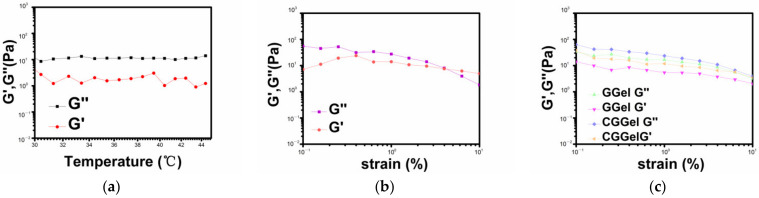
(**a**) The energy storage modulus G’) and loss modulus G″) of CGGel hydrogels at different temperatures. The energy storage modulus G’) and loss modulus G″) of Gel (**b**), GGel, and CGGel (**c**) hydrogels at different frequencies.

The rheological properties are crucial for hydrogels. It can analyze the stability and diffusion properties of hydrogels under different stresses [25]. We tested the rheology from 25 to 45 °C (Figure 5a), and the G″ of CGGel hydrogel is always higher than G’, which indicates that this hydrogel can have an excellent medical prospect (>37 °C). Meanwhile, we also measured the stability of three groups of hydrogels at different frequencies. We found that Gel hydrogels had an intersection of G’ and G″ at 6.31% (Figure 5b), while GGel and CGGel were always greater than G’ in the measurement range. The elastic modulus of CGGel was higher (Figure 5c), demonstrating that the addition of glycerol and AuNPs made the three-dimensional network-like structure of the hydrogels more robust, which improved the stability of the hydrogel and enhanced the application range.

#### 2.2.4. Microscopic Morphology of CGGel Gels

**Figure 6:**
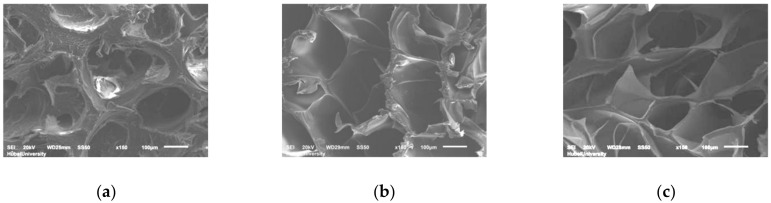
Scanning electron microscope (SEM) images of Gel (**a**), GGel (**b**), and CGGel (**c**) hydrogels. Bars represent 100 μm.

To further analyze the effect of glycerol and AuNPs addition on the microstructure of the hydrogels, we lyophilized the hydrogels and subsequently observed them by scanning electron microscopy (SEM). The 3-D mesh structure is one of the most significant properties of hydrogels and is the key to their optimal medical application [26]. The homogeneous and suitable pore size provides excellent water content, maintains a moist environment, and allows the exchange of gases, nutrients, and targeted release of drugs [27]. SEM showed the dense microporous structure and rough surface of the gelatin hydrogel (Figure 6a), owing to the self-crosslinking overdensity of the inherent triple helix structure of the highly concentrated gelatin hydrogel formed in the pure water environment during cooling formation. The addition of glycerol and AuNPs destroyed the tight network structure formed by the self-crosslinking of the spiral structure of gelatin (Figure 6b,c). Through the amino acid peptide chains in gelatin and the hydroxyl groups in glycerol, the reactive groups in AuNPs@Chlorella cross-link with each other, making the pores of the hydrogel larger and the surface smoother, which is more suitable for the needs of medical gels.

#### 2.2.5. Long-Lasting Moisture of CGGel Gels

**Figure 7:**
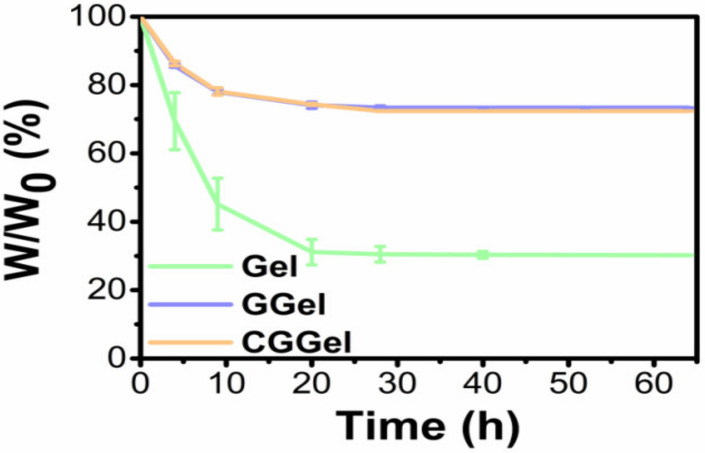
Weight loss plots of the hydrogels. Data are represented as the mean ± SD (*n* = 3).

Gelatin-based hydrogels have another disadvantage, a high dehydration rate. Glycerol is a common humectant used in various commercial medical dressings, skin care products, and food products [28]. In this study, the water retention of CGGel gels was evaluated by the weighting method (Figure 7). In an open environment, the water loss of pure aqueous gelatin gels decreased in a linear relationship (within 10 h). It maintained only 25% of its initial weight after one day, compared to GGel and CGGel hydrogels which maintained 70% of their initial weight when measured at the same time. The significant increase in water retention can be interpreted as the strong hydrogen bonds formed by the interaction between glycerol and water molecules, allowing a decrease in the water loss rate.

### 2.3. Antibacterial Activity of CGGel Gels

**Figure 8:**
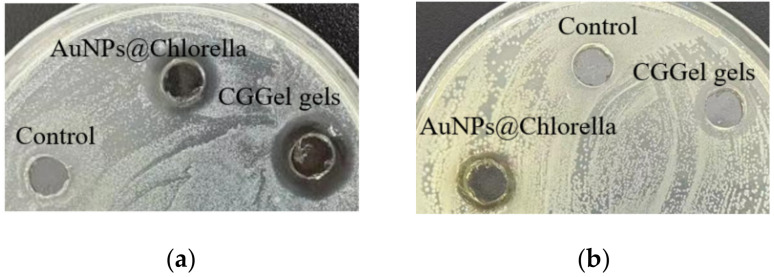
Photos showing *E. coli* (**a**) and *S. aureus* (**b**) zone of inhibition formation tests.

It is reported that the polyphenols and unsaturated fatty acid components of *Chlorella vulgaris* extracts have been shown to have some antibacterial properties (Figure 8) [29]. In this study, we evaluated the antibacterial ability of the hydrogels using the most typical *E. coli* and *S. aureus*. It was observed that blank control (water) showed no zone of inhibition on *E. coli* and *S. aureus* growth (incubated at 37 °C for 12 h), while AuNPs@Chlorella and CGGel hydrogels showed a clear zone of inhibition in both *S. aureus*. It could be observed that AuNPs@Chlorella and CGGel hydrogels induced more potent inhibition on *E. coli* growth (2.67 mm; 3.54 mm), as compared to that on *S. aureus* (1.53 mm; 1.71 mm) as indicated by the diameter of inhibition zone.

## 3. Conclusions

In summary, we successfully developed a new hydrogel (CGGel) with injectability, water retention, good stability, and antimicrobial properties through green and inexpensive materials, along with simple synthesis steps. AuNPs with good antimicrobial properties were synthesized using Chlorella extract as a reducing agent and loaded into gelatin–glycerol hydrogels (GGel), which improved the stability and phase transition temperature of GGel by cross-linking hydrogen bonds with each other while retaining the excellent injectability of gelatin and the water retention conferred by glycerol. The results demonstrated by our hydrogels are consistent with the properties required for an ideal dressing, which proposes new insights for future skin dressing design.

## 4. Materials and Methods

The following materials were used in this study: Gelatin, Tetrachloroauric acid (HAuCl_4_·3H_2_O), glycerin (China National Medicines, Beijing, China); Chlorella (Zhenghe Pharmaceutical, Shanxi, China) was procured from Zhenghe Pharmaceutical Co., Ltd. (Shanxi, China). *E. Coli* and *S. aureus* (Beina Biotech, Shanghai, China). Ultrapure water (Milli-Q grade, 18.2 MΩ cm; Millipore, Burlington, MA, USA) was used in all experiments.

### 4.1. Synthesis of AuNPs via the Green Method

A measure of 2 g of *Chlorella vulgaris* was added to 20 mL of deionized water at 80 °C for 4 h. After cooling, the extract was filtered through a 0.22 μL filter membrane to form a *Chlorella vulgaris* extract solution. Subsequently, the Chlorella extract solution was added to 0.01% HAuCl4 solution in the ratio of 1:10 (*v*/*v*). The mixture was magnetically stirred at 1000 rpm for 3 h in boiling water.

### 4.2. Preparation of Hydrogels

Gelatin (1.1 g) was completely dissolved in 3 mL ultrapure water/Gly solution/AuNPs solution in a different ratio, stirred well, and then left at room temperature to form Gel (3:0:0, *v*/*v*), GGel (1:1:0, *v*/*v*), and CGGel (2:1:1, *v*/*v*) hydrogels.

### 4.3. Physicochemical Characterization of AuNPs@Chlorella

The UV-Visible spectrum was used to determine the synthesis of AuNPs@Chlorella by measuring the maximum absorption values at wavelengths of 200–800 nm, and ultra-pure water was used as a blank; at the same time, the visual color transformation indicated the formation of AuNPs@Chlorella as well. X-ray powder diffraction (XRD) was used to analyze the crystalline phase and orientation properties of AuNPs@Chlorella with 2θ values in the range of 20–90° and a scan rate of 5°/min. Samples were washed 3 times using ultrapure water and subsequently freeze-dried before testing. The functional groups of AuNPs@Chlorella were analyzed by Fourier transform infrared spectroscopy (FTIR) and Raman spectroscopy, both in the test range of 0 cm^−1^ to 4500 cm^−1^. The AuNPs@Chlorella morphology was tested by transmission electron microscopy (TEM) with the AuNPs@Chlorella solution dropped on a 300-mesh copper grid and air-dried.

### 4.4. Characterisation of Hydrogels

#### 4.4.1. Determination of Gelation Time

The gelation time was determined using the vial inversion method by adding a volume of 1 mL of Gel, GGel, and CGGel to a glass vial (10 mL) at 37 °C and subsequently inverting every 30 s to determine the sol–gel time. The time that the gel was not flowing was recorded as the gelation time.

#### 4.4.2. The Retention Ability of the Hydrogel

The Gel, GGel, and CGGel hydrogels were tested in an open environment, and the weight of the hydrogels was recorded at several hourly intervals. The weight loss was calculated using the Equation (1) [30]:

Weight loss (%) = (W_0_ − W_t_)/W_0_ × 100%. (1)

W_0_ is the weight after a water loss, and W_t_ is the initial weight of the sample.

#### 4.4.3. The Adhesive Property of the Hydrogels

A qualitative macroscopic experiment was performed by injecting the hydrogel into the authors’ fingers and observing the adhesion at the joints with different angular movements.

#### 4.4.4. Scanning Electron Microscopy

Scanning electron microscopy was used to evaluate the network-like structure of the hydrogels. Gel, GGel, and CGGel were freeze-dried and then sprayed with platinum before testing.

### 4.5. In Vitro Antibacterial Activity Assay

For bacterial culture, solid or liquid Luria-Bertani (LB) medium was prepared. The solid LB medium was prepared as follows: tryptone (10 g), yeast extract (5 g), and sodium chloride (5 g) were fully dissolved in 950 mL of deionized water. The pH value of the solution was adjusted to 7, then added with agar powder (15 g). After that, the solution was diluted with deionized water to obtain a final volume of 1 L. After sterilization in an autoclave, the obtained LB medium was added to the plate for bacterial culture (15 mL per plate). The liquid LB medium was prepared following the same procedure without agar powder.

The synthesized samples were evaluated for their antibacterial activity against *E. coli* and *S. aureus* using the agar well diffusion method [31]. Briefly, *E. coli* (BNCC 336 953) and *S. aureus* (BNCC 186 335) were incubated in the liquid LB medium overnight at 37 °C. 20 µL of *E. coli*/*S. aureus* (concentration 1.0 × 10^7^) were evenly spread on the plates, followed by injecting equal volumes of water, AuNPs@Chlorella, and CGGel, and then incubated in an incubator at 37 °C for 12 h. At the end of the incubation period, the zone of inhibition was quantified by measuring the zone diameter (Three parallel samples for each group, n = 3).

## Data Availability

The data that support the findings of this study are available from the corresponding author upon reasonable request.
